# Supplementary material for: Photodegradation Process of Organic Dyes in the Presence of a Manganese-Doped Zinc Sulfide Nanowire Photocatalyst
Source: Materials (Basel). 2021 Oct 6;14(19):5840. doi: 10.3390/ma14195840 (PMC8510231; doi:10.3390/ma14195840)
Supplement: Supplementary file 1 [file materials-14-05840-s001.zip › materials-1343453-supplementary.pdf]

materials

Supplementary Materials

Photodegradation Process of Organic Dyes in The Presence of a Manganese Doped Zinc Sulfide Nanowire Photocatalyst

Adam Żaba, Svitlana Sovinska, Tetiana Kirish, Adam Węgrzynowicz and Katarzyna Matras-Postołek *

Faculty of Chemical Engineering and Technology, Cracow University of Technology, Warszawska 24, 31-155 Cracow, Poland; adam.p.zaba@gmail.com (A.Ż.), svitlanastorm@mail.ru (S.S.), tanyakirish@gmail.com (T.K.), adam.wegrzynowicz@pk.edu.pl (A.W.)

* Correspondence: k.matras@pk.edu.pl; Tel.: + 48 126283059

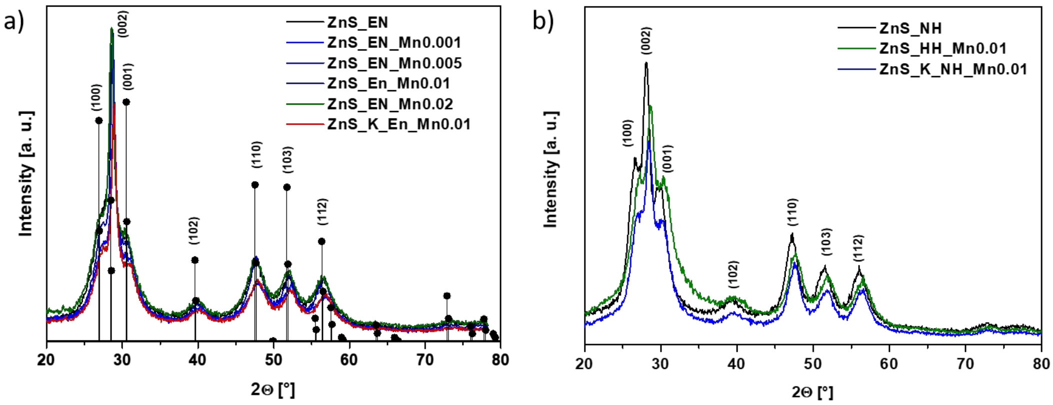

Figure S1. XRD patterns (**a**) of ZnS nanocrystals stabilized with ethylenediamine and (**b**) ZnS nanocrystals stabilized with hydrazine; the peaks in the pattern matching well with wurtzite structure according to JCPDS#96-110-0045.

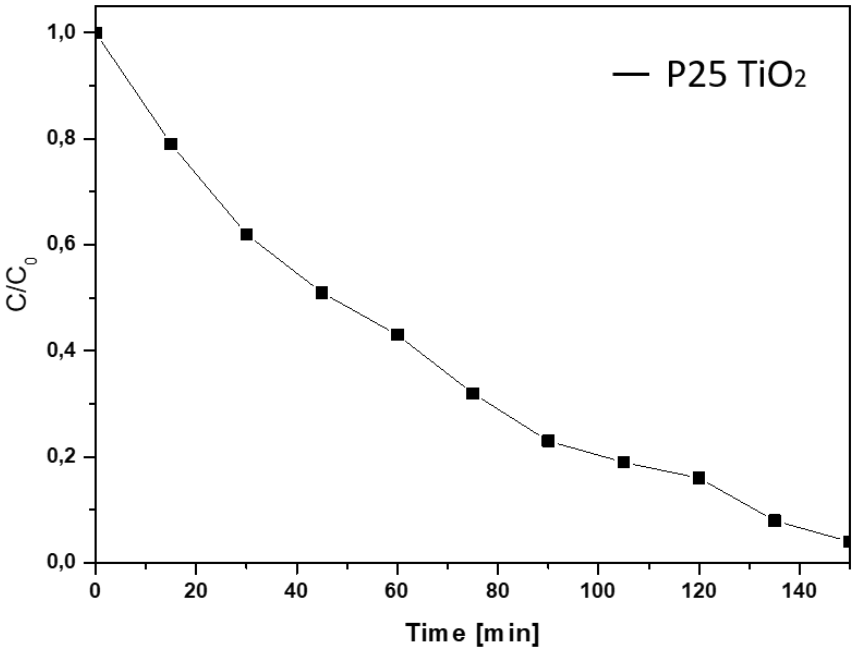

Figure S2. Time-dependent UV-Vis absorption spectra for RhB observed during incubation with the P25 TiO_2 samples under UV illumination.
